# Treatment of Muckle-Wells syndrome: analysis of two IL-1-blocking regimens

**DOI:** 10.1186/ar4237

**Published:** 2013-05-29

**Authors:** Jasmin B Kuemmerle-Deschner, Helmut Wittkowski, Pascal N Tyrrell, Ina Koetter, Peter Lohse, Katharina Ummenhofer, Fabian Reess, Sandra Hansmann, Assen Koitschev, Christoph Deuter, Anja Bialkowski, Dirk Foell, Susanne M Benseler

**Affiliations:** 1 Division of Pediatric Rheumatology, Department of Pediatrics, University Hospital Tuebingen, Germany; 2 Department of General Pediatrics, University Children's Hospital Muenster, Germany; 3 Division of Rheumatology, Department of Pediatrics, The Hospital for Sick Children, University of Toronto, Canada; 4 Division of Rheumatology, Department of Internal Medicine, University Hospital Tuebingen, Germany; 5 Institut fuer Labormedizin, Prof. Blessing, Bereich Molekularmedizin-Singen, Germany; 6 Pediatric Otorhinolaryngology, Klinikum Stuttgart-Olgahospital, Germany; 7 Centre for Ophthalmology, University Hospital Tuebingen, Germany; 8 Institute of Immunology, University of Muenster, Germany

**Keywords:** *NLRP3*, *CIAS1*, mutation, Muckle-Wells syndrome, autoinflammatory fever syndromes, interleukin-1 inhibition, anakinra, canakinumab, S100A12

## Abstract

**Objectives:**

Muckle-Wells syndrome (MWS) is an autoinflammatory disease characterized by excessive interleukin-1 (IL-1) release, resulting in recurrent fevers, sensorineural hearing loss, and amyloidosis. IL-1 inhibition with anakinra, an IL-1 receptor antagonist, improves clinical symptoms and inflammatory markers. Subclinical disease activity is commonly observed. Canakinumab, a fully human IgG1 anti-IL-1β monoclonal antibody, can abolish excess IL-1β. The study aim was to analyze the efficacy and safety of these two anti-IL-1 therapies.

**Methods:**

Two cohorts of patients with severe MWS and confirmed *NLRP3 *mutation were treated with anakinra and/or canakinumab. Clinical and laboratory features including ESR, CRP, SAA, and the neutrophil marker S100A12 were determined serially. Disease activity was captured by MWS disease activity scores (MWS-DAS). Remission was defined as MWS-DAS ≤5 plus normal CRP and SAA. Treatment efficacy and safety were analyzed.

**Results:**

The study included 12 anakinra- and 14 canakinumab-treated patients; the median age was 33.5 years (3.0 years to 72.0 years); 57% were female patients. Both treatment regimens led to a significant reduction of clinical disease activity and inflammatory markers. At last follow-up, 75% of anakinra-treated and 93% of canakinumab-treated patients achieved remission. During follow-up, S100A12 levels mirrored recurrence of disease activity. Both treatment regimens had favorable safety profiles.

**Conclusions:**

IL-1 blockade is an effective and safe treatment in MWS patients. MWS-DAS in combination with MWS inflammatory markers provides an excellent monitoring tool set. Canakinumab led to a sustained control of disease activity even after secondary failure of anakinra therapy. S100A12 may be a sensitive marker to detect subclinical disease activity.

## Introduction

Muckle-Wells syndrome (MWS) is an autoinflammatory disease in the spectrum of inherited cryopyrin-associated periodic syndromes (CAPS). CAPS comprise the mild familial cold-induced autoinflammatory syndrome (FCAS), the moderate MWS, and the severe neonatal-onset multisystem inflammatory disease (NOMID), also known as chronic infantile neurologic, cutaneous, articular (CINCA) syndrome [1-3]. Most CAPS patients carry mutations in the *NLRP3 *gene encoding the protein cryopyrin/NALP3 [4,5] which is essential for the activation of intracellular caspase 1 and the processing of interleukin-1β (IL-1β) [6-11]. Macrophages from MWS patients show a constitutive increase of IL-1β [2,10,12,13].

Excessive production of IL-1β in MWS patients leads to attacks of fever, rash, musculoskeletal symptoms, and conjunctivitis. These characteristic features occur episodically and can last between 1 day and 2 weeks. Musculoskeletal symptoms include arthralgia, arthritis, and significant myalgia. Urticarial rash and amyloidosis, together with progressive sensorineural hearing loss, are clinical findings supporting the diagnosis of MWS [14]. Severe fatigue is commonly found and has a significant impact on the quality of life of MWS patients. Sequelae of MWS include progressive sensorineural hearing loss, ultimately leading to deafness, and renal amyloidosis.

Inflammatory markers, including C-reactive protein (CRP) and the erythrocyte sedimentation rate (ESR), are commonly elevated in patients with MWS, particularly during acute inflammatory episodes [15]. Serum amyloid A (SAA) is a marker of neutrophil activation and inflammation. In patients with amyloidosis, SAA has been reported to predict risk of mortality [16].

The neutrophil activation marker S100A12 (also named EN-RAGE and calgranulin C) is secreted by granulocytes [17], binds to the receptor for advanced glycation end products (RAGE), and shows a strong pro-inflammatory activity [17,18]. High S100A12 levels have been found in the serum of active systemic juvenile idiopathic arthritis (sJIA) and familial Mediterranean fever (FMF) patients [19-21].

Treatment of MWS patients targets IL-1. Studies supported the efficacy of IL-1 inhibition with either rilonacept, a dimeric fusion protein consisting of the ligand-binding domains of the extracellular portions of the IL-1 receptor components (IL-1 TRAP) [22,23], canakinumab [24], or anakinra [25]. Anakinra is a recombinant, soluble, nonglycosylated IL-1 receptor antagonist (IL-1Ra) [15,26-30] and blocks the biologic activity of IL-1 by competitively binding to the IL-1 type I receptor (IL-1RI) expressed on a wide variety of tissues [31]. Anakinra therapy leads to symptom control in patients with CAPS [26]. However, frequent high-dose injections are not well tolerated [29,30]. Canakinumab, a fully human IgG1 anti-IL-1β monoclonal antibody, has been shown to provide selective and sustained blockade of IL-1β, neutralizing the effect of excess IL-1β. Canakinumab is reported to be well tolerated with no infusion-related adverse events and no formation of anti-canakinumab antibodies [24].

The aims of this study were (a) to report the clinical and laboratory features of MWS patients requiring IL-1 blockade, (b) to determine the impact of IL-1 blockade with either anakinra or canakinumab on clinical features and laboratory markers, and (c) to analyze the efficacy and safety of the two IL-1-blocking therapies in patients with MWS.

## Methods

### Study design

A single-center open-label, prospective observational study of consecutive pediatric and adult patients diagnosed with active MWS between April 2004 and August 2008 was performed. All patients were treated with anakinra and/or canakinumab. Informed individual consent was obtained from all patients for *NLRP3 *mutation testing and for off-label and experimental treatment. Approval from the local ethics committee (Ethik Kommission der Medizinischen Fakultät der Universität Tübingen) was obtained (REB no. 325/2007 BO1).

### Patients

MWS patients were eligible if they met the following criteria: (a) clinical features of active MWS requiring medical intervention and (b) genetic confirmation of *NLRP3 *mutation, as previously described [32]. Patients were excluded, if they (a) were concurrently treated with other immune-modulatory therapies such as methotrexate, (b) were younger than 3 or older than 76 years of age at enrollment, (c) had evidence of a preexisting underlying infection or significant medical conditions including heart disease, recent infections, drug or alcohol abuse, or (d) had received live vaccinations within the past 3 months. Female patients of child-bearing age were required to start an effective method of contraception.

Patients were followed according to a standardized protocol in the institutional autoinflammatory clinics by experienced pediatric and adult rheumatologists (JKD, IK). Clinical and laboratory features and disease-activity scores were collected from serial standardized assessments obtained for all patients.

Data from some of the patients examined here have been used in previous publications [24,30,32,33].

#### Anakinra cohort

Patients received anakinra (Kineret; Amgen, Cambridge, UK) at a dose of 1 to 2 mg/kg/day in patients <40 kg body weight and at a dose of 100 mg/day for those of ≥40 kg body weight. The drug was self-administered by subcutaneous injection once daily. In children with persistent disease activity, the anakinra dose was stepwise escalated to a maximum of 8 mg/kg. Concurrent nonsteroidal antiinflammatory medication was added, if required.

#### Canakinumab cohort

Patients were treated with canakinumab at a dose of 2 mg/kg SC for <40 kg body weight or 150 mg SC for ≥40 kg body weight every 8 weeks. In patients who did not achieve remission by day 8, canakinumab was administered at a dose of 5 mg/kg body weight intravenously. Patients were allowed to switch anti-IL-1 therapy for lack of efficacy or because of patient preference (roll-over). On discontinuation of anakinra treatment, a disease flare had to be awaited. The maximum wait time before the start of canakinumab therapy for recurrence of disease activity (flare) was limited to 14 days.

### Demographics and clinical characteristics

Demographic information included gender, ethnicity, age at diagnosis, age at onset of IL-1 blockade, and duration of follow-up. Standardized assessments reviewed constitutional symptoms of fever (pattern and duration) and fatigue and MWS organ manifestations, including headache, ocular symptoms including conjunctivitis, uveitis, papilledema, other eye symptoms, sensorineural hearing loss, oral ulcers, abdominal pain, arthralgia, arthritis, myalgia, and skin symptoms, including erythematous and cold-induced rash. Associated conditions, disease complications, and sequelae, including hearing impairment or loss, renal impairment, and delayed puberty, were recorded.

### Muckle-Wells Syndrome Disease Activity Score (MWS-DAS)

The previously developed MWS-DAS determines disease activity in nine MWS domains attributing 0, 1, or 2 points to each level of disease activity [32]. MWS-DAS captures (a) fever, (b) headache, (c) eye disease, (d) hearing impairment, (e) oral ulcers, (f) abdominal pain, (g) renal disease, (h) musculoskeletal disease, and (i) rash. The tenth domain is the Patient Global Assessment score measured on a visual analogue scale (VAS) and categorized into 0 points for ≤1 cm, 1 point for >1 to 5 cm, and 2 points for strictly >5 to 10 cm. A MWS-DAS of <10 points was considered mild disease activity. Scores ≥10 points indicated severe MWS disease activity, as previously described [32]. Remission was defined as inactive disease reflected in a MWS-DAS ≤5 plus normalized CRP and SAA.

### Inflammatory markers

Standardized laboratory testing was performed at each visit and included ESR, CRP, total white blood count (WBC), neutrophil count, hemoglobin (HGB), platelet count (PLT), SAA, and S100A12. The latter was determined in patient sera by a double sandwich enzyme-linked immunosorbent assay (ELISA) system, as described previously [34].

### Study end points and outcome

The primary study end points (short-term end points) were day 14 of anakinra and day 8 of canakinumab therapy, based on the distinct pharmacokinetics of each IL-1-blocking drug. Long-term end point was the patient's last follow-up visit. All patients were treated with either therapy until last follow-up. Treatment efficacy was measured at each end point, including the percentage of patients achieving remission, clinical disease activity as determined by MWS-DAS, and inflammatory markers. Treatment safety was recorded and included mild and severe infections; the latter was defined as requiring hospitalization, injection-site reactions, and all other possible adverse events.

### Analysis

Baseline characteristics were compared by using descriptive statistics. Means and standard deviations, or medians and ranges, for nonparametric variables, were reported. Student *t *test was used for continuous parametric variables, and χ^2 ^and Fisher exact test for categoric variables. The paired *t *test was used for parametric variables, and Wilcoxon signed-rank test for nonparametric repeated measurements on single samples. Correlations were analyzed by using the Spearman rho test. All *P *values less than 0.05 were considered significant. All analyses were performed by using SAS for Windows, version 9.2 (SAS Institute Inc., Cary, NC, USA).

## Results

### Patients

In total, 21 patients with the clinical diagnosis of MWS and confirmatory evidence of a *NLRP3 *mutation were screened for inclusion into the study. Patients came from four different families. Nine were males and 12, females. All patients were Caucasians. The median age at diagnosis of MWS was 33.5 (range, 3.0 to 72.0 years). Six patients were 18 years of age or older.

#### Anakinra cohort

Eight patients did not meet the inclusion criteria because of minimally active disease. One patient was receiving dialysis and was excluded.

#### Canakinumab cohort

Four patients were excluded because of dialysis (one), multiple sclerosis (one), immunosuppression after renal transplant (one), and colon cancer (one). Three patients did not consent to canakinumab therapy.

### Baseline demographics

#### Anakinra cohort

In total, 12 patients (three males and nine females) were included, of whom seven were adults, and five, children. The median age was 19.0 years (range, 3.0 to 65.5 years) at MWS diagnosis and 21.0 years (range, 3.0 to 66.5 years) at start of anakinra therapy. Three different *NLRP3 *gene mutations were found. Seven (58%) patients carried the E311K mutation. The remaining mutations included T348 M in three (25%) and V198M in two (17%) patients. Median follow-up at the long-term end point was 52 months (range, 23 to 115 months). All patients had severe MWS (MWS-DAS ≥10).

#### Canakinumab cohort

In total, 14 patients (five males and nine females) were included, of whom eight were adults, and six, children. The median age was 27.0 years (range, 3.0 to 72.0 years) at MWS diagnosis and 29.0 years (range, 4.0 to 74.5 years) at onset of canakinumab therapy. *NLRP3 *gene mutations were E311K in 10 (71%), T348 M in two (14%), and V198M in two (14%) patients. Median follow-up at the long-term end point was 50 months (range, 23 to 110 months). The MWS disease activity at diagnosis was severe in 10 (71%) and mild in four (29%).

##### Rollover

The 10 patients with severe disease at diagnosis had previously received anakinra. These were two males and eight females with a median age of 20.0 years (4.0 to 47.2 years) at the start of canakinumab therapy. Five of the 10 patients were children. Three children switched to canakinumab for secondary treatment failure despite anakinra dose escalation and excellent treatment adherence. The mutations seen in these were E311K in one and V198M in two children. The remaining two children and five adult rollover patients started canakinumab for personal preference. The *NLRP3 *gene mutations were E311K in six (60%), T348 M in two (20%), and V198M in two patients (20%). The mutations of the rollover children were V198M in two and E311K in one. All 10 had severe disease activity at the time of treatment initiation.

### Disease activity

At baseline, the mean MWS-DAS was 13 in the anakinra cohort, reflecting severe disease. At the primary study end point, the MWS-DAS decreased significantly to 3 (*P *< 0.001). At the last follow-up, the mean MWS-DAS was 4, which is still significantly decreased when compared with baseline (*P *< 0.001). The canakinumab cohort had a mean MWS-DAS of 6 at baseline, which significantly decreased to 3 at the primary end point (*P *= 0.002). At last follow-up, the mean disease activity further decreased to 2, remaining significantly reduced from baseline (*P *< 0.001).

### Inflammatory markers

#### Anakinra cohort

At baseline, 92% had abnormal CRP and SAA levels. ESR was elevated in 75%; 55% had elevated S100A12 levels, including one patient with high clinical disease activity but normal CRP and SAA levels. At 14 days of anakinra therapy, the mean ESR decreased significantly from 9 at baseline to 1 mm/hour (*P *= 0.02). The mean S100A12 levels were statistical significantly lower than at baseline (*P *= 0.05). At last follow-up, ESR, CRP, and SAA levels were significantly lower than at baseline (all *P *< 0.01). In contrast, the mean S100A12 level increased, in parallel to the mild increase in disease activity measured by MWS-DAS. All data are summarized in Table 2.

**Table 1:** Characteristics of patients with Muckle-Wells syndrome (MWS) treated with interleukin-1-blocking therapy

	Anakinra cohort *n *= 12	Canakinumab cohort *n *= 14
Demographics		

Male/female patients	3:9	5:9

% females	75	64

Median age at MWS diagnosis in years (range)	19.0 (3.0 to 65.5)	27.0 (3.0 to 72)

Median age at anti IL-1 therapy in years (range)	21.0 (3.0 to 66.5)	29.0 (4.0 to 74.5)

*NLRP3 *mutation		

Mutation subtypes: E311K No (%) T348M No (%) V198M No (%)	7/12 (58%) 3/12 (25%) 2/12 (17%)	10/14 (71%) 2/14 (14%) 2/14 (14%)

Follow-up		

Median follow-up in months (range)	52 (23 to 115)	50 (23 to 110)

**Table 2:** Treatment efficacy of anakinra and canakinumab in patients with MWS

IL-1 inhibitor	Anakinra cohort (*n *= 12)					Canakinumab cohort *(n *= 14)				
	Baseline	Short-term end point^a^	Comparison short-term end point, baseline	Long-term end point^b^	Comparison long-term end point, baseline	Baseline	Short-term end point^a^	Com-parison short-term end point, baseline	Long-term end point^b^	Com-parison long-term end point, baseline

**Disease activity score (MWS-DAS)**										

Mean MWS-DAS (stdv)	13 (2.2)	3 (1.0)	**0.0005**	4 (3.2)	**0.0005**	6 (1.9)	3 (1.8)	**0.002**	2 (1.3)	**0.0002**

**Inflammatory markers**										

Mean ESR in mm/h (stdv)	32 (17)	13 (16)	**0.02**	10 (5)	**0.0005**	24 (9.8)	11.9 (4.6)	**<0.0001**	8.4 (4.8)	**<0.0001**

Elevated ESR (%) (>22 mm/h)	9 (75)	1 (8)	-	1 (8)	-	9 (64)	0		1 (7)	

Mean CRP in mg/dl (stdv)	2.1 (1.3)	0.9 (1.9)	0.09 NS	0.4 (0.5)	**0.0005**	2.3 (2.1)	0.1 (0.1)	**0.001**	0.2 (0.3)	**0.001**

Elevated CRP (%) (>0.5 mg/dl)	11 (92)	4 (33)	-	3 (25)	-	12 (86)	0	-	1 (7)	-

Mean SAA in mg/L (stdv)	36.5 (26.1)	27.5 (70.5)	0.67 NS	6.6 (5.2)	**0.001**	88.4 (185.1)	3.4 (3.8)	**0.01**	4.50 (5.8)	0.11 NS

Elevated SAA (%) (>10 mg/L)	11 (92)	4 (33)	-	3 (25)	-	9 (64)	1 (7)	-	1 (7)	-

Mean S100A12 in ng/ml (stdv)	240 (172) (11/12)	142 (57) (11/12)	**0.05**	273 (330) (11/12)	0.70 NS	284.1 (255.5)	73.4 (51.4)	**0.01**	76.4 (44.5)	**0.01**

Elevated S100A12 (%) (>130 ng/ml)	6/11 (55)	6/11 (55)	-	5/11 (45)	-	10 (71)	1 (7)	-	2 (14)	-

**Remission (MWS-DAS ≤5 plus normal CRP and SAA)**										

Patients in remission (%)	NA	8 (67)		9 (75)		NA	13 (93)		13 (93)	

Stdv, standard deviation. ^a^Short-term end point was 14 days for anakinra and 8 days for canakinumab, based on the specific pharmacokinetic features of each drug. ^b^Median long-term end point was 12 (5 to 14) months for anakinra and 11 (6 to 15) months for canakinumab.

#### Canakinumab cohort

At baseline, 86% of the patients had elevated CRP, and 64% had increased SAA levels; 71% had elevated S100A12 levels, and a high ESR was seen in 64%. All patients with raised SAA also had elevated CRP levels. At the primary end point, none of the patients had CRP or ESR abnormalities (*P *< 0.01); one continued to have abnormal SAA (*P *= 0.01) and S100A12 levels (*P *= 0.01). At last follow-up, mean inflammatory marker levels remained normal in the majority of patients (ESR, CRP, and SAA in 93% and S100A12 in 86%). All levels except SAA were statistically significantly lower than baseline. All results are summarized in Table 2.

### Comparison of canakinumab and anakinra

**Figure 1:**
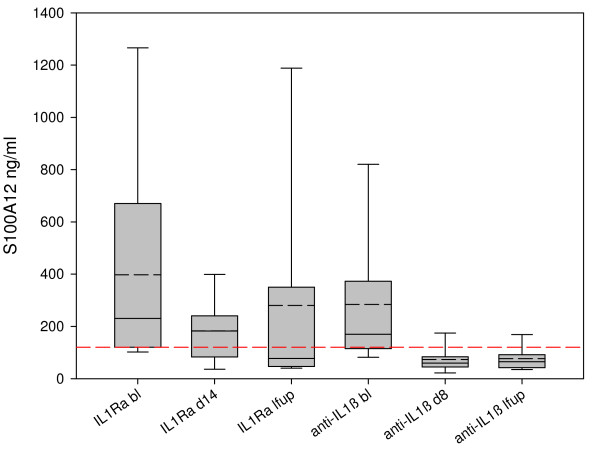
**S100A12 levels in Muckle-Wells syndrome (MWS) patients, comparing interleukin-1 blockade with anakinra (IL-1Ra) and canakinumab (anti-IL-1β)**. Serum S100A12 levels were measured in MWS patients treated with anakinra (IL-1Ra) at baseline (IL-1Ra bl) and at the short-term end point (IL-1Ra d14) and at last follow-up (IL-1Ra lfup). Similarly levels were determined in patients treated with canakinumab (anti-IL-1β) at baseline (anti-IL-1β bl) and at the short-term end point (anti-IL-1β d8) and at last follow-up (anti-IL-1β lfup). Box plot depicts median, mean (dashed line), 25^th ^and 75^th ^percentiles (box) and 10^th ^and 90^th ^percentiles. S100A12 levels are reduced by anakinra both at short- and long-term end points. Treatment with canakinumab reduced S100A12 levels even further and below the limits of normal (red dashed line).

Both anti-IL-1 regimens were efficacious in controlling clinical and laboratory disease activity. The short-term response rates for both treatments were excellent. Anakinra-treated patients had high clinical baseline disease-activity scores, which were significantly improved at the primary end point (*P *= 0.0005). In contrast, patients receiving canakinumab had overall lower baseline MWS-DAS scores. The mean disease activity similarly significantly improved (*P *= 0.002), including in patients previously treated with anakinra. Inflammatory markers improved with both treatment regimens. Patients treated with canakinumab had a sustained improvement at last follow-up (see Table 2). S100A12 levels over the course of the study period are depicted in Figure 1.

A total of 67% of anakinra-treated patients achieved clinical and laboratory remission and had no evidence of active disease at day 14; 75% remained in remission at the long-term study end point. Three of the five pediatric patients required dose escalation, whereas none of the adult patients did. In canakinumab-treated patients, disease remission was documented in 93% at both study end points. No detectable difference in treatment efficacy was found when comparing IL-1-blockade naïve and rollover patients treated with canakinumab. Within the rollover group, those treated for secondary treatment failure to anakinra and those switched for personal preference also had a similar canakinumab-treatment effect. In addition, in children and adults, canakinumab therapy was effective. One patient not achieving remission at last follow-up was an adult.

### Treatment safety

#### Anakinra cohort

No serious adverse events were observed. Mild adverse events included injection-site reactions in five (42%) as well as weight gain of ≥5 kg and mild upper respiratory infections in four (33%) patients, respectively.

#### Canakinumab cohort

One patient developed vertigo, which was considered a serious adverse event because of the requirement for hospital admission. The vertigo self-resolved, and the patient continued on canakinumab therapy. No injection-site reactions were seen. Mild upper respiratory tract symptoms were recorded in four (29%) and transient headache in two (14%).

## Discussion

The study evaluated the short- and long-term efficacy of two distinct regimens of IL-1 blockade in pediatric and adult MWS patients with active disease. The high disease activity at baseline, as measured by the MWS-DAS score, the inflammatory markers ESR, CRP, SAA, and the neutrophil activation marker S100A12 was significantly reduced by both IL-1-inhibiting regimens.

Ninety-three percent of canakinumab-treated patients achieved remission at the short-term and the long-term end points. Canakinumab led to a statistically significant reduction of CRP, ESR, SAA, and S100A12 levels into the normal range. The clinical disease activity decreased further at last follow-up, indicating stable and sustained remission. Canakinumab had an excellent safety profile in this study, in agreement with efficacy and safety data recently documented in CAPS patients [24,35].

Sixty-seven percent of anakinra-treated patients achieved remission at the short-term end point, and 75%, at the long-term study evaluation. Only ESR and S100A12 levels decreased significantly at 2 weeks, paralleling the MWS-DAS at this study end point. However, at last follow-up, the disease activity increased slightly, mirroring the increase in S100A12 levels in some patients. In contrast, mean CRP, ESR, and SAA further decreased. The anakinra safety profile was good. However, injection-site reactions were a major complaint, similar to other reports [15].

Disease activity in MWS is best captured when combining clinical and laboratory parameters. Our study used the MWS-DAS to determine clinical disease activity. The measure was responsive to change after anti-IL-1 treatment. Inflammatory markers mirrored MWS disease activity. With clinical improvement ESR, CRP, SAA, and S100A12 levels decreased. ESR is a widely available test and has been used in previous anti-IL-1 treatment studies [15,23]. CRP and SAA are increasingly used at international centers to monitor the course of diseases of the innate immune system. Both have been found to be sensitive markers of active FMF [36]. SAA is a predictive marker for poor outcome in patients with amyloidosis. Lachmann [16] calculated a fivefold increased risk of death associated with high SAA levels. S100A12 was shown to be overexpressed at sites of local inflammation [37]. The neutrophil activation marker was found at very high levels in sJIA and FMF [20]. In sJIA patients with inactive disease, increasing S100A12 levels indicated flares as early as weeks before becoming clinically apparent [19].

The study had several limitations. Overall, it included only a small number of children and adults with MWS. However, this is one of the largest prospective cohorts of MWS patients published to date, in whom four laboratory parameters and standardized clinical disease-activity measures were systematically studied while monitoring two distinct anti-IL-1 treatment regimens. The study was not a randomized controlled trial. The study design and the inclusion of rollover patients included a high risk for confounding by indication. Overall disease activity was significantly higher in the anakinra cohort compared with the canakinumab cohort at baseline. The study included treatment-naïve and pretreated patients, some of whom only had a partial response to initial anti-IL-1 therapy, despite dose escalation. We documented that these patients, who were then considered secondary treatment failures to anakinra, responded well to canakinumab.

## Conclusions

IL-1-blocking therapies led to a significant clinical improvement and normalization of inflammatory markers in the majority of MWS patients. All but one patient treated with canakinumab achieved complete and sustained remission, whereas one fourth of anakinra-treated patients demonstrated features of subclinical inflammatory activity returning at last follow-up. We suggest that switching to canakinumab after secondary failure to anakinra may be a therapeutic option in MWS.

## Abbreviations

Anti-IL-1β bl: canakinumab treatment at baseline; anti-IL-1β lfup: canakinumab treatment at last follow-up; CAPS: cryopyrin-associated periodic syndrome; CIAS1: cold-induced autoinflammatory syndrome 1; CINCA: chronic infantile neurologic cutaneous and articular syndrome; CRP: c-reactive protein; ELISA: enzyme-linked immunosorbent assay; ESR: erythrocyte sedimentation rate; FCAS: familial cold autoinflammatory syndrome; FMF: familial Mediterranean fever; HGB: hemoglobin; IgG1: immunoglobulin G1; IL-1: interleukin-1; IL-1β: interleukin-1-beta; IL-1RA: IL-1-receptor antagonist; IL-1Ra bl: IL-1-receptor antagonist treatment at baseline; IL-1Ra lfup: IL-1-receptor antagonist treatment at last follow-up; IL-1RI: IL-1-receptor type 1; MWS: Muckle-Wells syndrome; MWS-DAS: Muckle-Wells syndrome Disease Activity Score; NLRP3/NALP3: NOD-like receptor family, pyrin domain containing 3; NOMID: neonatal onset, multisystem inflammatory disease; PLT: platelet count; S100A12: also named EN-RAGE and calgranulin; SAA: serum amyloid A; sJIA: systemic juvenile idiopathic arthritis.

## Conflict of interest

JKD performed clinical studies with and received honoraria from Novartis. The other authors declare that they have no competing interests in respect to this study.

## Authors' contributions

All authors read and approved the final manuscript. JKD, HW, DF, and SMB conceived of the trial and planned the data analysis, wrote and revised the manuscript. PNT performed the statistical analysis. PL did the genetic analysis. AK did the audiologic examinations and data analysis. CD did the ophthalmologic examinations. IK, KU, FR, SH, and AB performed the rheumatologic examinations, clinical and laboratory data gathering, and analysis.
